# Editorial: Precision therapeutics using next generation technologies in transplantation

**DOI:** 10.3389/frtra.2024.1371701

**Published:** 2024-03-07

**Authors:** Satish N. Nadig, Joseph Leventhal, Lorenzo Gallon, Carl Atkinson

**Affiliations:** ^1^ Department of Surgery, Microbiology/Immunology, and Pediatrics, Comprehensive Transplant Center, Feinberg School of Medicine, Northwestern Medicine, Northwestern University, Chicago, IL, United States; ^2^ Division of Transplant, University of Illinois, Chicago, IL, United States

**Keywords:** transplant, immunology, proteomics, allocation, perfusion

**Editorial on the Research Topic** Precision therapeutics using next generation technologies in transplantation

The field of transplantation today is in the midst of a *renaissance*. The future of transplantation will clearly rely on next generation technologies to allow for more targeted drug delivery approaches, sophisticated cellular isolation techniques, organ repair technologies, advanced diagnostic techniques, and use of tissues originating from regenerative technological approaches. This Research Topic is dedicated to the introduction of such next generation approaches to personalize transplantation beyond the broad applications that currently constitute the standard of care.

In this unique topic review, discussions span cellular therapies such as a) use of xeno-based islet transplantation using porcine islets while overcoming immunologic barriers b) using single-cell RNA-sequencing (scRNA-seq) technology to discern between donor and recipient origin and c) the use of high-definition novel “top-down” proteomics to identify biomarker and therapeutic targets in transplantation. These novel approaches are essential and ***the time is now***.

The current state of transplant continues to utilize systemic immunosuppression identified over 3 decades ago, preservation solutions developed in the 1980s, and there are still no precise ways to pre-treat or condition organs prior to transplant. These realities persist as the “gold standard” (1, 2). Yet, disparities in organ availability and recipients in need are wide, morbidity and mortality of systemic immunosuppression continue without any progress in the rate of graft failure due to chronic rejection, and a lack of targeted therapeutics and diagnostics prior to organ implantation continues to hinder optimal organ-recipient matching (3, 4). Science, however, has progressed with gene editing technologies and nanoscale approaches to theranostics; therefore, the time is ripe for the field of transplantation to enter the ***next era of technology***. The authors of the manuscripts in this Research Topic recognize this need and, through their own work, summations, or personal viewpoints, suggest a biopsy of methods where new technological applications are directed to solid organ transplantation (SOT). Here, we summarize some of the salient points raised in each of these articles and discuss ways the field may advance.

The common adage that “xenotransplantation is around the corner, *and always will be*” may, now more than ever, be called into question. The last couple of years have seen porcine kidneys used in the decedent model and CRISPR-edited pig hearts transplanted into humans (5, 6). Although the clinical success of these paradigm shifting feats were limited, the science behind the xeno transplants indeed moved the field forward. The first article in our series by Eisenson et al. takes a more granular approach to xenotransplantation by reviewing the use of islet transplantation using porcine islets and tackles the various hurdles within the innate and adaptive immune systems that lie in the way of successful islet engraftment- namely, instant blood mediated inflammatory reactions (IBMIR). Specifically, they discuss stealth encapsulation of islets to prevent IBMIR, along with transgenic pig development to grow islets with carbohydrates more compatible with human tissue engraftment. Tolerance induction strategies including chimerism approaches along with the combined infusion of regulatory T cells are also discussed. Finally, a novel method of targeting diabetic nephropathy with combined islet/kidney thymic tissue engraftment is introduced.

The Research Topic spans solid orgna transplant above and below the diaphragm with the second article focusing on the use of Glucagon-like peptide-1 (GLP-1) agonism with Atrial Natriuretic Peptide (ANP) as a biomarker in *ex vivo* pre conditioning of lungs using a large animal porcine model. These unique pretreatment strategies challenge the current paradigm of preservation alone. With the advent of machine perfusion and more targeted therapeutic approaches, the preservation phase of organ transplantation can be utilized not only to extend the life of a graft but also to expand the donor pool to the use of marginal organs with various reparative strategies. In fact, Whitson and Black explore this on a more global scale in their opinion piece, where they examine the ethical considerations surrounding the establishment of “organ assessment and repair centers (ARC).” The who, what, and why around the supervision and operations of potential ARCs are explored, setting the seed for the future of organ-intensive care units to improve organ recipient matching. The ramifications of such pre conditioning strategies go beyond the organ itself and have direct implications on the allocation system and waitlist beyond its current form (7, 8).

The Research Topic continues with a deep dive into next generation tools that will most certainly alter the way we manage the complex immunologic milieu of the organ and the host. We have historically focused broadly on the adaptive system for immunosuppression; yet, here, we attempt to more specifically target innate responses (9, 10). Ott and Cuenca look at the interplay of the innate system and provide insights on how best to stymie early responses to provide better long-term outcomes. In fact, the immunologic origins are more specifically delineated by Wilson et al. with single-cell RNA-sequencing (scRNA-seq) technologies identifying the origins of cellular subsets to specifically manipulate the immune system in a more precise and personalized way. Finally, novel “top-down” proteomic technologies are introduced by Huang et al., and implement the use of specific mass spectrometry on whole tissues to drill down to isolated proteoforms to asses for organ quality and markers for long term graft function. These proteoforms identified from tissue biopsies can add to the library of biomarkers which may be called upon in real time. Top-down proteomic methodology, importantly, will allow for new generation therapeutic and drug development.

It is an exciting time to be part of solid organ transplantation community. Indeed, the future is now, and concerted efforts must be made to break down silos in order to pave the path forward. Machine perfusion, for example, should synergize with nanotherapeutics for organ pre conditioning. Organ procurement organizations need a more integrated approach with academic medical centers for organ rehabilitation which will inform allocation policies. Cellular therapeutic and regenerative medicine options must exist in concert with academic and engineering clean cell facilities. These are just a few examples of the untapped potential that exists in the field of transplantation today.
